# Identification of Novel HIV-1 Latency-Reversing Agents from a Library of Marine Natural Products

**DOI:** 10.3390/v10070348

**Published:** 2018-06-27

**Authors:** Khumoekae Richard, David E. Williams, E. Dilip de Silva, Mark A. Brockman, Zabrina L. Brumme, Raymond J. Andersen, Ian Tietjen

**Affiliations:** 1 Faculty of Health Sciences, Simon Fraser University, Burnaby, BC V5A 1S6, Canada; kra43@sfu.ca (K.R.); mark_brockman@sfu.ca (M.A.B.); zbrumme@sfu.ca (Z.L.B.); 2 Departments of Chemistry and Earth, Ocean & Atmospheric Sciences, University of British Columbia, Vancouver, BC V6T 1Z4, Canada; davewill@chem.ubc.ca (D.E.W.); raymond.andersen@ubc.ca (R.J.A.); 3 Department of Chemistry, University of Colombo, Colombo 03, Sri Lanka; edilip.desilva@gmail.com; 4 Department of Molecular Biology and Biochemistry, Simon Fraser University, Burnaby, BC V5A 1S6, Canada; 5 British Columbia Centre for Excellence in HIV/AIDS, Vancouver, BC V6Z 1Y6, Canada

**Keywords:** HIV-1, latency reversal, HIV reservoir, natural products, antivirals, shock-and-kill, psammaplin A, aplysiatoxin, debromoaplysiatoxin, alotaketal C

## Abstract

Natural products originating from marine and plant materials are a rich source of chemical diversity and unique antimicrobials. Using an established *in vitro* model of HIV-1 latency, we screened 257 pure compounds from a marine natural product library and identified 4 (psammaplin A, aplysiatoxin, debromoaplysiatoxin, and previously-described alotaketal C) that induced expression of latent HIV-1 provirus in both cell line and primary cell models. Notably, aplysiatoxin induced similar levels of HIV-1 expression as prostratin but at up to 900-fold lower concentrations and without substantial effects on cell viability. Psammaplin A enhanced HIV-1 expression synergistically when treated in combination with the protein kinase C (PKC) activator prostratin, but not the histone deacetylase inhibitor (HDACi) panobinostat, suggesting that psammaplin A functions as a latency-reversing agent (LRA) of the HDACi class. Conversely, aplysiatoxin and debromoaplysiatoxin synergized with panobinostat but not prostratin, suggesting that they function as PKC activators. Our study identifies new compounds from previously untested marine natural products and adds to the repertoire of LRAs that can inform therapeutic “shock-and-kill”-based strategies to eliminate latent HIV-infected reservoirs.

While current licensed HIV-1 therapies inhibit virus replication, they do not act on latently-infected CD4+ T cells which have HIV-1 provirus incorporated within their genomes. As these proviruses can reactivate at any time to produce infectious virus, novel approaches are needed to eliminate these HIV-1 reservoirs [1,2,3]. One therapeutic strategy, frequently termed “shock-and-kill” [4], proposes the treatment of latent HIV-infected cells with latency-reversing agents (LRAs) to induce proviral expression (“shock”), after which these cells may be eliminated by viral cytopathic effects or host immune responses (“kill”). Numerous LRAs have been identified that belong to distinct functional classes, primarily histone deacetylase inhibitors (HDACi) and protein kinase C (PKC) activators [5,6]. However, while several of these LRAs reproducibly induce HIV-1 proviral expression *in vitro* and/or *in vivo*, to date no LRA has appreciably reduced the size of the inducible viral reservoir in clinical trials, underscoring a need for new LRAs with improved efficacy [5,6,7]. Toward this goal, several studies show that treatment of cells with combinations of LRAs from distinct functional classes yields synergistic responses that are significantly greater than the expected additive effects of these agents [8,9,10,11,12,13,14,15]. However, as outcomes from clinical trials with LRA combinations are not yet reported, continued discovery of additional LRAs and LRA combinations remains a priority.

Natural products obtained from plant and marine sources are a rich source of diverse chemical compounds, including HIV-1 inhibitors and novel LRAs [16,17]. Moreover, screens for novel LRAs from natural product libraries often result in “hit” rates of 1.0% or more [18,19], indicating that even small natural chemical libraries may be sufficient to identify new agents of interest. Here, we describe the results from a screen for novel LRAs from a library of 257 pure and structurally diverse natural compounds derived from marine invertebrates and microorganisms. These compounds were assembled over many years by the laboratory of R.J.A. (e.g., [20,21,22,23,24]); however, many of these compounds have no known molecular targets or have only been reported to possess basic cytotoxic or antimicrobial activity. We previously screened this library for compounds that inhibit HIV replication and identified 6 with 50% effective concentrations (EC_50_s) of 3.8 µM or less, including at least one (bengamide A) that acts by mechanisms which are distinct from licensed antiretrovirals [25]. Here we describe 4 new LRAs identified from this library, including 1 HDACi and 3 PKC activators.

All natural compounds were confirmed ≥ 95% pure by NMR and LC/MS and dissolved in DMSO to stock concentrations of 5 mg/mL. The control LRA TNFα (Sigma-Aldrich, Oakville, ON, Canada) was dissolved in PBS plus 0.1% bovine serum albumin to a stock concentration of 5 µg/mL. The control LRA panobinostat (HDACi; Sigma-Aldrich) was dissolved in DMSO to a stock concentration of 30 mM. The control LRA prostratin (PKC activator; Selleck Chemicals, Houston, TX, USA) was dissolved in PBS to a stock concentration of 1.5 mM.

J-Lat Full Length T cell lines (clones 8.4, 9.2, and 10.6) were obtained from the NIH AIDS Reagent Program, Division of AIDS, NIAID, NIH (contributed by Dr. Eric Verdin) [26]. These cells are derived from Jurkat cells and contain a transcriptionally-silent, HIV-1 proviral genome encoding a frameshift mutation within Env, and where Nef is replaced with a GFP reporter [26]. As a result, GFP expression in these cells indicates reactivation of HIV-1 from latency. Cells were maintained in R10+ medium (RPMI 1640 with HEPES and L-Glutamine (Lonza, Mississauga, ON, Canada), 10% fetal calf serum, 100 U/mL penicillin, and 100 µg/mL streptomycin (Sigma-Aldrich)) at 37 °C and 5% CO_2_.

For latency-reversal assays, J-Lat cells were re-suspended in R10+ medium to a concentration of 10^6^ cells/mL. 2 × 10^5^ cells were then seeded into 96-well plate wells with compounds or control LRAs at desired final concentrations. Control cells were incubated at a final concentration of up to 0.3% DMSO vehicle control. In no case did final test concentrations of DMSO exceed 0.3%, which had no observable effects on cell viability or GFP expression. Cells were incubated at 37 °C and 5% CO_2_ for 24 h, after which a minimum of 5000 cells from each culture was analyzed by flow cytometry (Guava EasyCyte 8HT, EMD Millipore, Etobicoke, ON, Canada). Flow cytometry data were analyzed using FlowJo v. 8.8.7 software (FlowJo LLC, Ashland, OR, USA), and results were reported as the mean ± s.e.m. from at least 3 independent experiments.

Compounds were initially assessed in J-Lat 9.2 cell cultures based on observations from us and others that this cell line responds to LRAs from multiple functional classes but rarely achieves GFP expression in all cells, thereby enabling comparative dose-response studies across large concentration ranges in addition to accurate studies of synergistic effects of LRA combinations [12,14,15]. Representative flow cytometry data using this cell line and control LRAs are shown in Figure 1A. For each experiment, control J-Lat cell cultures treated with 0.1% DMSO were first gated for typical forward and side-scatter profiles consistent with viable, healthy cells [14,15]. This subset was then assessed for GFP expression across all cell cultures, with the GFP-positive gate set such that the average of GFP-positive cells in DMSO-treated control cell cultures was 0.05% (Figure 1A, top). For each culture treated with compound, the extent of latency reversal was then measured as the percentage of GFP-positive cells. For example, in one representative experiment, treatment of J-Lat cells with 0.1 µg/mL panobinostat (~0.15 µM) resulted in 13.9% GFP-positive cells, while treatment with 10 µg/mL prostratin (~12 µM) resulted in 6.2% GFP-positive cells (Figure 1A, center and bottom). Consistent with published data [8,15,27], both control LRAs exhibited dose-dependent expression of GFP across multiple concentrations (Figure 1B). For example, maximal GFP expression in J-Lat cells was observed after treatment with 1.4 µM panobinostat (17.1 ± 2.7%) or 12 µM prostratin (6.4 ± 0.9%) (Figure 1B). Using the approach of Hashemi et al. [28] and normalizing results relative to the average GFP response for 12 µM prostratin (the concentration at which maximal prostratin activity was observed), the EC_50_s for panobinostat and prostratin were calculated to be 0.10 ± 0.02 and 7.1 ± 2.8 µM, respectively (Table 1). These results confirm that panobinostat is approximately 70-fold more potent than prostratin, which is also consistent with previous reports [8,15,27].

To directly assess the impact of control LRAs on cell viability (i.e., in a manner independent of provirus expression), parental Jurkat cells (Clone E6-1, American Type Culture Collection; Manassas, VA, USA), which do not harbor integrated HIV-1 provirus, were prepared and treated with LRAs as described above. Following 24 h incubation, compound toxicity was assessed by measuring surface expression of the early apoptotic marker annexin V by flow cytometry (by staining with annexin V-APC; BioLegend, San Diego, CA, USA). Results were reported as the fold-increase in annexin V-positive cells relative to 0.1% DMSO-treated control cells (mean ± s.e.m.) from at least 3 independent experiments. Representative data are shown in Figure 1C. Here, treatment with 0.1% DMSO resulted in 13.6% of cells with APC-fluorescence above the bulk of the cell population, which is presumed to lack surface expression of annexin V (top). In contrast, treatment with 0.15 µM panobinostat resulted in 58.6% APC-positive cells (i.e., a 4.3-fold increase in apoptosis from DMSO control) (center), while 12 µM prostratin resulted in 24.5% APC-positive cells (i.e., 1.8-fold increase from DMSO control) (bottom). As expected, control LRAs increased cellular apoptosis in a dose-dependent manner (Figure 1D). For example, treatment with 0.045 µM panobinostat induced a 5.8 ± 1.2-fold increase in apoptosis, indicating poor cellular tolerance at concentrations that induced latency reversal, while at least 10 µM prostratin induced no more than a 2.0 ± 0.3-fold increase in apoptosis (Figure 1D). Taken together, our control experiments confirm that panobinostat is a more potent, yet also more toxic, LRA than prostratin [8,15,27].

We next screened 257 structurally-diverse pure compounds derived from marine natural products at 2.5 µg/mL for latency reversal activity in J-Lat 9.2 cells. Of these, seven (2.7%) compounds resulted in cytolysis and disruption of cell morphology as observed by light microscopy, consistent with widespread cell death, and were not considered further. Of the remaining 250 compounds, four induced GFP expression in at least 4% of cells (Figure 2A). This “hit” rate of 1.6% is in line with previously reported screens of natural product libraries (~0.5–1.1%) [18,19] and supports the notion that pure natural product libraries are enriched for bioactive LRAs compared to synthetic small molecule libraries, where reported hit rates of ~0.1% are more frequent [28,35,36].

Structures of the four identified compounds are shown in Figure 2B. Notably, psammaplin A, originally isolated from the two-sponge associate *Poecillastra* sp. and *Jaspis* sp. [29], was previously identified as an HDACi with anti-tumor activity [30,31]. In contrast, aplysiatoxin and debromoaplysiatoxin, which differs from aplysiatoxin by the loss of a bromine atom in the phenol ring, are toxins produced by blue-green algae and potent PKC activators [32,33,34]. However, not all HDACis and PKC activators possess potent HIV latency modulatory functions [14,37,38], and none of these compounds have been investigated as HIV-1 LRAs. Finally, alotaketal C, originally isolated from *Phorbas* sp., is a potent activator of cyclic AMP and PKC signaling that we recently characterized for its HIV-1 latency reversal activity [14,20] and is thus not assessed further here.

Each new LRA induced dose-dependent reversal of HIV-1 latency in J-Lat cells across multiple concentrations (Figure 2C,D). The average maximum responses observed for each compound ranged from 1.4 to 2.7-fold more than controls treated with 12 µM prostratin. For example, psammaplin A induced GFP expression in up to 17.6 ± 4.0% of cells at 5 µg/mL (3.8 µM). When results were normalized to the average GFP response for 12 µM prostratin [28], psammaplin A’s EC_50_ was calculated as 1.9 ± 0.3 µM, approximately 19-fold higher than the EC_50_ of panobinostat (Table 1). In contrast, aplysiatoxin induced GFP expression in 9.4 ± 0.1% cells with as little as 0.15 µg/mL (0.1 µM) and yielded a calculated EC_50_ of 0.045 ± 0.021 µM. Aplysiatoxin is therefore 160-fold more potent than prostratin and 2.2-fold more than panobinostat, identifying it as a particularly potent LRA in J-Lat 9.2 cells. Debromoaplysiatoxin induced GFP expression in 7.2 ± 1.5% of cells at 1.5 µg/mL (1.3 µM) and yielded a calculated EC_50_ of 0.92 ± 0.14 µM, or 7.7-fold more potent than prostratin.

In Jurkat cells, we observed that 3.8 µM psammaplin A induced a 3.3 ± 0.6-fold increase in annexin V staining, with extensive cell death observed at higher concentrations. In contrast, no more than 2.0 ± 0.2 and 1.6 ± 0.1-fold increases in annexin V staining were observed for aplysiatoxin and debromoaplysiatoxin, respectively (Figure 2E,F). Thus, psammaplin A appeared to be toxic at concentrations that induced latency reversal, while both aplysiatoxin and debromoaplysiatoxin were largely well-tolerated across all concentrations.

These agents displayed similar dose-response profiles in J-Lat 8.4 cells, indicating that they act on HIV provirus independent of its integration site (Figure 3A). For example, when results were normalized to the average GFP response for 38 µM prostratin (i.e., the concentration at which maximal activity was observed in J-Lat 8.4 cells), the EC_50_ of psammaplin A was calculated as 1.5 ± 0.1 µM, or approximately 20.5-fold higher than the EC_50_ of panobinostat (Table 1). Aplysiatoxin induced detectable GFP expression at concentrations as low as 0.0001 µM, with a calculated EC_50_ of 0.011 ± 0.003 µM, or 900-fold more potent than prostratin and 6.6-fold more than panobinostat. Similarly, an EC_50_ of 0.52 ± 0.02 µM was calculated for debromoaplysiatoxin, or 19.2-fold more potent than prostratin.

In J-Lat 10.6 cells, we observed that all LRAs were capable of inducing GFP expression in at least two-thirds of cells (Figure 3B), indicating robust efficacy. However, we also observed an average of 7.5% of J-Lat 10.6 cells spontaneously expressing GFP in the absence of LRAs, consistent with previous reports [39], indicating a lower barrier to HIV latency reversal compared to J-Lat 9.2 and 8.4. Nevertheless, when results were normalized to the average GFP response for 3.8 µM prostratin (one of three concentrations where maximum activity was observed), the EC_50_ of psammaplin A was again calculated as 1.5 ± 0.1 µM, or approximately 36.6-fold higher than the EC_50_ of panobinostat (Table 1). Moreover, GFP expression was observed with aplysiatoxin concentrations as low as 3.7 × 10^−5^ µM (37 pM) and a calculated EC_50_ of 0.0033 ± 0.0012 µM, or 540-fold more potent than prostratin and 12.4-fold more than panobinostat. Finally, the calculated EC_50_ of debromoaplysiatoxin (0.081 ± 0.029 µM) was 22.2-fold more potent than prostratin. Thus, the rank-order of potency for all LRAs was consistent across all cell lines.

To confirm that LRAs induce HIV protein expression in addition to the GFP reporter, J-Lat 10.6 cells were also stained with the HIV-1 p24^Gag^ antibody KC57-RD1 (Beckman Coulter, Indianapolis, IN, USA) and processed using the Cytofix/Cytoperm Fixation/Permeabilization Kit (BD Biosciences, Mississauga, ON, Canada) prior to flow cytometric analysis. Results were then reported as the fold-increase in p24^Gag^-positive cells relative to 0.1% DMSO-treated control cells (mean ± s.e.m.) from at least 3 independent experiments. All LRAs were observed to induce at least 9.7-fold increased p24^Gag^-positive cells, with the same rank order as observed for GFP expression (Figure 3C). This confirms that LRAs also induce viral protein expression.

To investigate whether LRAs induce proviral expression in primary human cells, we obtained peripheral blood mononuclear cells (PBMCs) from three HIV-infected donors on stably-suppressive antiretroviral therapy for at least three years (Figure 4). Study protocols were approved by the Institutional Review Boards of Simon Fraser University and the University of British Columbia—Providence Health Care Research Institute (REB: H15-03077, approved 8 March 2016). Written informed consent was obtained from all donors. Frozen PBMC aliquots were thawed and allowed to recover in R10+ medium at 37 °C, 5% CO_2_ for 24 h at 2.5 × 10^6^ cells/mL. PBMCs were then incubated at 10^6^ cells/mL with positive control cell activators PMA (100 ng/mL) plus ionomycin (1 µg/mL), 3.8 µM psammaplin A, 1.1 µM aplysiatoxin, 1.3 µM debromoaplysiatoxin, or 0.1% DMSO vehicle control. All conditions were performed in duplicate. Following 24 h incubation at 37 °C and 5% CO_2_, supernatant p24^Gag^ protein was quantified by ELISA (Xpress Bio, Frederick, MD, USA), and cell viability was measured by flow cytometry using Guava Viacount, a DNA intercalating dye (EMD Millipore). In most cases, each LRA caused an increase in supernatant p24^Gag^ above background; however, substantial donor-to-donor variation was observed, consistent with other studies [10,11,12] (Figure 4A). For example, while treatment with PMA + ionomycin induced an average 62.3 ± 31.1% (mean ± s.e.m.) increase in supernatant p24^Gag^ relative to untreated cells, psammaplin A resulted in a 85.0 ± 41.6% increase. Similarly, aplysiatoxin and debromoaplysiatoxin induced increases of 56.0 ± 15.0 and 46.9 ± 22.3%, respectively. No major changes in cell viability were observed, with a maximum 15.4 ± 2.2% (mean ± s.e.m.) reduction in viability in the presence of 3.8 µM psammaplin A (Figure 4B). These results indicate that LRAs have the capacity to activate latent HIV-1 provirus in primary human cells isolated from persons with long-term viremia suppression on antiretroviral therapy.

As described previously [8,9,10,11,12,13,14,15], treatment of cells with combinations of LRAs acting through different mechanisms tends to result in synergistic effects on HIV-1 latency reversal, while treatment with compounds acting through similar mechanisms tends to yield at most additive responses. These observations can therefore be used to identify potential functional classes of novel LRAs [14,15]. To demonstrate this, we assessed GFP expression in J-Lat 9.2 cells treated with novel LRAs in combination with control LRAs, including the pro-inflammatory cytokine TNFα, the HDACi panobinostat, and the PKC activator prostratin (Figure 5). In these studies, synergism was observed in all cases where control LRAs were applied in combination: for example, treatment of J-Lat 9.2 cells separately with 0.01 µg/mL TNFα or 0.15 µM panobinostat induced 22.3 ± 2.5% and 8.9 ± 1.8% GFP-positive cells, respectively, whereas treatment of cells with both compounds simultaneously led to 49.0 ± 1.2% GFP-positive cells, which is ~1.6-fold higher than would be expected if the effects of these two compounds were strictly additive (i.e., 31.2%; Figure 5). Similarly, treatment with TNFα plus 12 µM prostratin, or panobinostat plus prostratin, induced responses that were 1.6- and 2.6-fold higher than expected for additive effects, respectively. These levels of synergism were statistically significant (*p* < 0.05; Student’s unpaired *t*-test with a two-sided, Bonferroni correction) as measured by the Bliss independence model, which was calculated as described previously [10,11].

Treatment of J-Lat 9.2 cells with 2 µM psammaplin A in addition to TNFα or prostratin induced 49.8 ± 1.1 and 44.4 ± 5.1% GFP-positive cells, representing 1.7- and 2.7-fold increases in GFP expression over what would be expected from strictly additive effects, respectively. However, treatment of J-Lat cells with psammaplin A plus panobinostat induced only 9.8 ± 1.7% GFP-positive cells, or 0.6-fold of what would be expected by strictly additive effects (Figure 5). These results are consistent with the known function of psammaplin A as a HDACi [30,31]. Conversely, treatment of cells with 2 µM aplysiatoxin plus TNFα or panobinostat induced 51.1 ± 0.5 and 48.4 ± 2.2% GFP-positive cells, respectively, or 1.5- and 2.3-fold increases in GFP-positive cells over expected additive effects, while co-treatment with prostratin induced only 12.2 ± 1.8% GFP-positive cells, or 0.6-fold of expected additive effects. These observations are consistent with the known function of aplysiatoxin as a PKC activator [33,34]. Similar results were found using debromoaplysiatoxin: co-treatment with TNFα or panobinostat induced 50.4 ± 1.5 and 47.6 ± 1.5% GFP-positive cells, respectively, or 1.5 or 2.3-fold over expected additive effects, while co-treatment with prostratin induced 12.0 ± 1.8% GFP-positive cells, or 0.6-fold of expected additive effects, indicating that its latency reversal activity is also likely due to activation of PKC. All synergistic effects were statistically significant as measured with the Bliss independence model. Taken together, these results indicate that the latency reversal properties of these pure natural products in J-Lat cells are consistent with their previously reported functional properties.

In summary, we identify four LRAs derived from marine natural products that can be added to the repertoire of known HIV-1 shock-and-kill agents. The likely mechanisms of action for all four compounds, supported here and by prior studies, are consistent with established functional classes, including one HDACi (psammaplin A) and three PKC activators (aplysiatoxin, debromoaplysiatoxin, and previously-described alotaketal C) [14]. Dose response profiles suggest that psammaplin A is a less potent LRA than panobinostat, while aplysiatoxin and debromoaplysiatoxin are more potent than prostratin. These observations were confirmed in two additional J-Lat cell lines and in PBMCs from three HIV-infected donors, indicating that latency reversal occurs independent of proviral integration sites and that these compounds can reverse latency in primary cells. The contributions of these mechanisms to HIV-1 latency reversal were further supported by synergism studies, described both here and elsewhere [14].

Our previous discovery of multiple novel HIV inhibitors from this chemically-diverse library (e.g., [25]) led us to hypothesize that we might also identify new modulators of HIV latency with distinct molecular mechanisms. However, while this screen instead identified only compounds of the HDACi and PKC activation classes, the results described here should not preclude future screening of natural product-derived compound libraries for additional LRAs which may act by novel mechanisms of action. Conversely, as the kinetic properties of both HDACis and PKC activators do not necessarily correspond with latency reversal efficacy [14,37,38], testing of library compounds with both known and unknown molecular functions remains warranted. In support of this, we notably identify one PKC activator, aplysiatoxin, that is particularly potent across multiple cell models, with activity observed in J-Lat 8.4 and 10.6 cells at picomolar concentrations. Finally, we note that additional latency reversal mechanisms by these agents may remain undetected. For example, we previously described alotaketal C as an LRA of the PKC activator class [14], but it is additionally reported to function as an activator of cyclic AMP signaling [20], which may also modulate HIV-1 latency reversal [40,41].

Taken together, this study highlights the benefits of natural product-based screens for LRA discovery. The identification and evaluation of new LRAs will support the development of novel therapeutic combinations and clinical approaches to reduce or eliminate latent HIV-1 reservoirs.

## Figures and Tables

**Figure 1:**
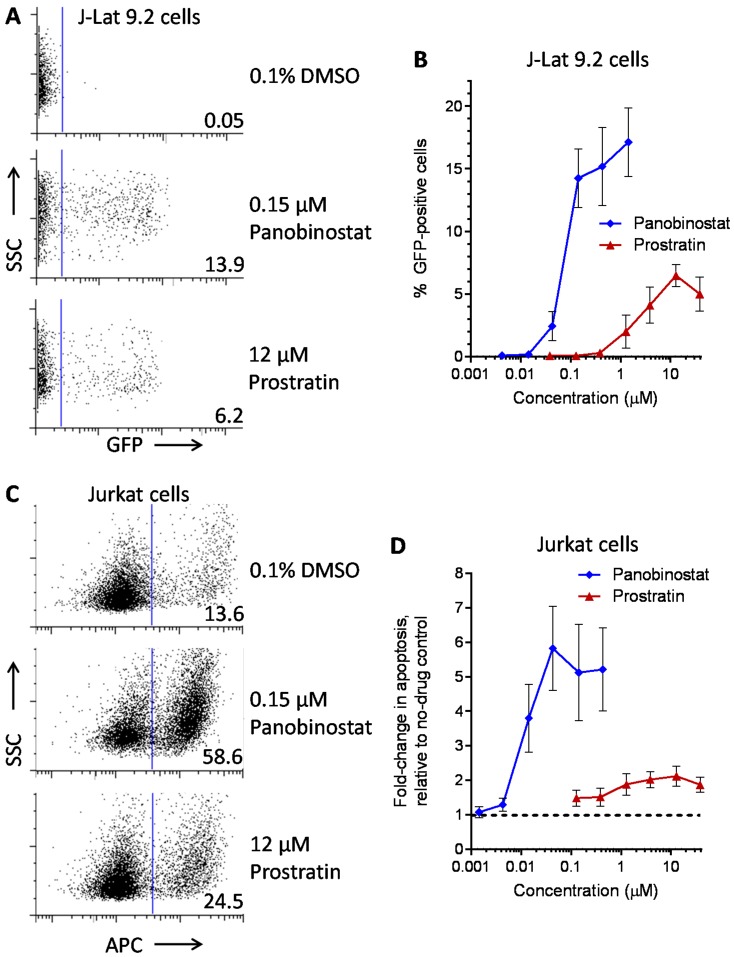
HIV-1 latency reversal and cellular toxicity assessments of control LRAs. (**A**) Representative GFP expression in J-Lat cells treated with 0.1% DMSO (top), 0.15 µM panobinostat (center), or 12 µM prostratin (bottom). Numbers to the right of the blue bar indicate percent GFP-positive (i.e., HIV-expressing) cells. (**B**) Effects of control LRAs on HIV-1 provirus expression in J-Lat cells. (**C**) Representative effects of compounds on apoptosis in Jurkat cells, as measured by annexin V staining. Numbers to the right of the blue bar indicate percent APC-positive (i.e., apoptotic) cells. (**D**) Effects of control LRAs on apoptosis in Jurkat cells, expressed as fold-increase in apoptosis relative to control cultures treated with 0.1% DMSO (dotted line).

**Figure 2:**
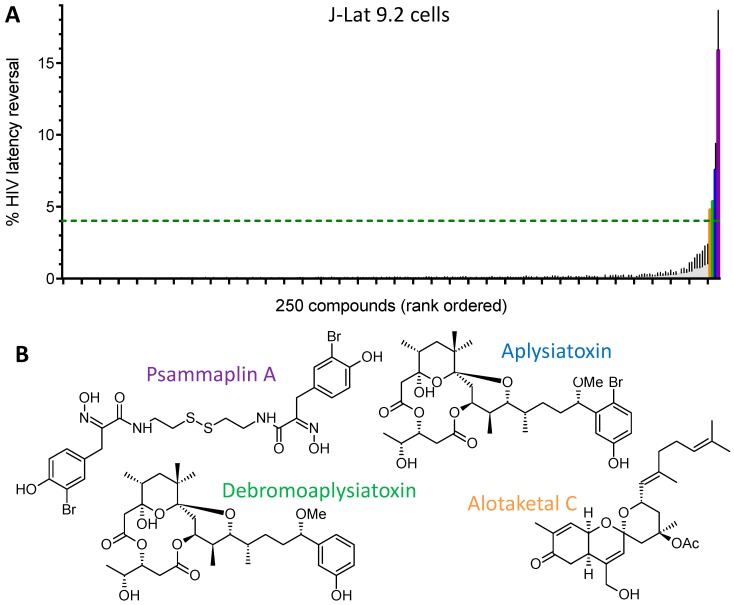

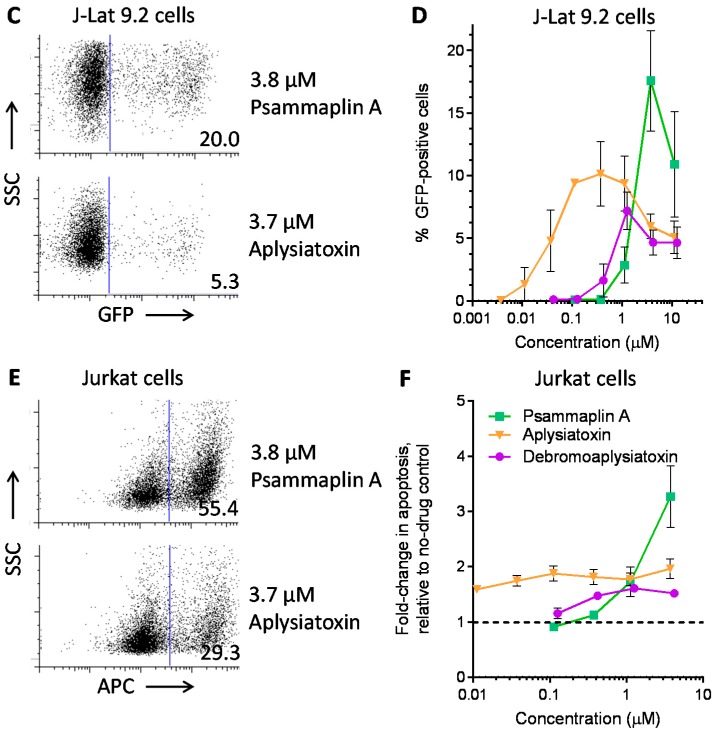
Discovery of new LRAs from marine natural products. (**A**) HIV latency reversal in J-Lat 9.2 cells, as assessed by measuring GFP reporter expression in the presence of 250 compounds from marine natural products at 2.5 µg/mL. Colored bars denote 4 of 250 compounds (1.6%) that induced GFP expression in at least 4% of cells (dotted line). (**B**) Structures of 4 LRAs identified from screening of 257 pure natural products at 2.5 µg/mL. Colors correspond to bars in panel A. (**C**) Representative GFP expression in J-Lat 9.2 cells treated with 3.8 µM psammaplin A (top) or 3.7 µM aplysiatoxin (bottom). Numbers to the right of the blue bar indicate percent GFP-positive (i.e., HIV-expressing) cells. (**D**) Effects of LRAs on HIV-1 provirus expression in J-Lat cells. (**E**) Representative effects of compounds on apoptosis in Jurkat cells, as measured by annexin V staining. Numbers to the right of the blue bar indicate percent APC-positive (i.e., apoptotic) cells. (**F**) Effects on apoptosis in Jurkat cells, expressed as fold-increase in apoptosis relative to control cultures treated with 0.1% DMSO (dotted line). Data presented in panels C and E originate from the same experiments shown in Figure 1A,C, respectively.

**Figure 3:**
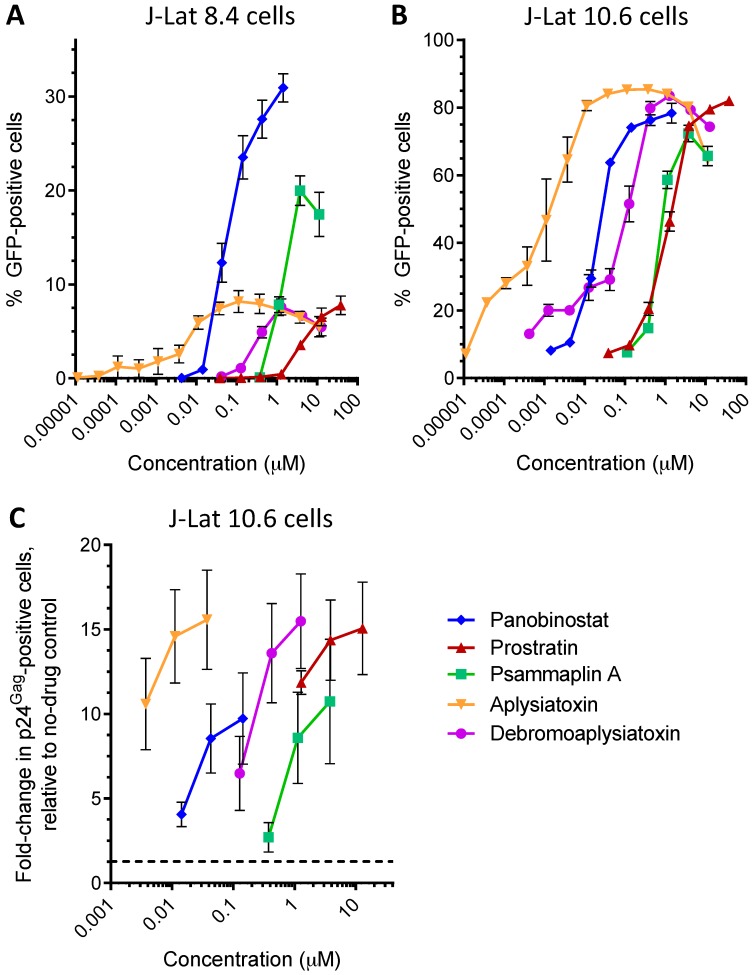
Natural products reverse latency in multiple cell line models. (**A**,**B**) Effects of LRAs on GFP expression in J-Lat 8.4 (**A**) and 10.6 cells (**B**). (**C**) Effects of LRAs on intracellular viral p24^Gag^ expression in J-Lat 10.6 cells. Data are presented as fold-change in supernatant p24^Gag^ relative to cells treated with 0.1% DMSO vehicle control (dotted line).

**Figure 4:**
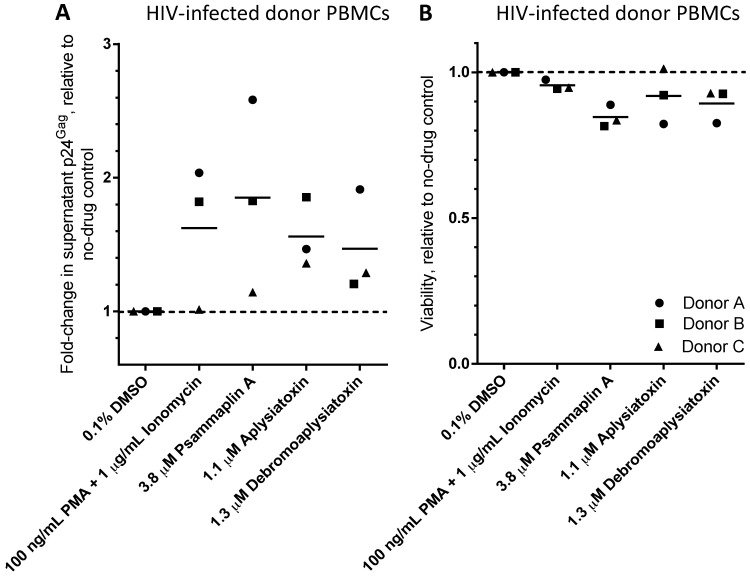
Effects of LRAs on peripheral blood mononuclear cells (PBMCs) from three HIV-infected donors. (**A**) p24^Gag^ viral protein levels in PBMC culture supernatants following 24 h treatment with control compounds or candidate LRAs for 24 h. Data are presented as fold-change in supernatant p24^Gag^ relative to PBMCs treated with 0.1% DMSO vehicle control (dotted line). (**B**) Viability of PBMCs after 24 h treatment with LRAs, as measured by Viacount cell-permeable dye. Data are presented as viability relative to PBMCs treated with 0.1% DMSO vehicle control (dotted line). In both panels, shapes represent PBMCs from individual donors.

**Figure 5:**
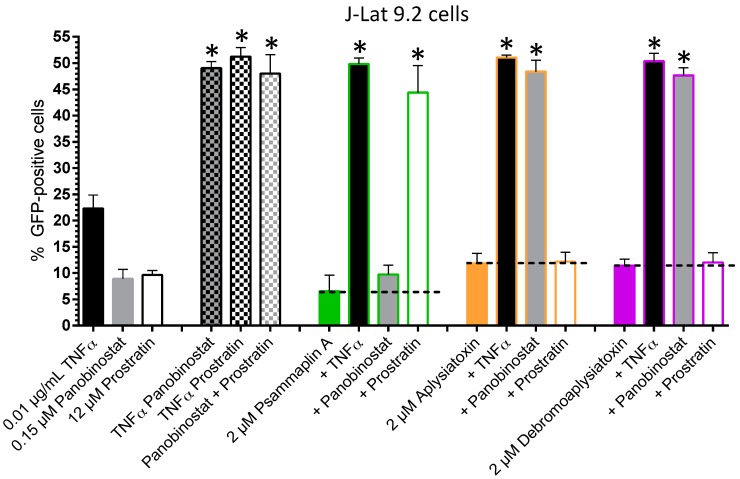
Effects of LRAs and LRA combinations on HIV-1 provirus expression in J-Lat cells. * *p* < 0.05 using the Bliss independence model [10,11].

**Table 1:** 50% effective concentrations (EC_50_s) of latency-reversing agents (LRAs). EC_50_s were calculated in J-Lat 9.2, 8.4, and 10.6 cells based on the percent of GFP expression relative to controls treated with 12, 38, or 3.8 µM prostratin, respectively, using the approach of Hashemi et al. [28]. n.d., not determined.

LRA	EC50 (µM; mean ± s.e.m.)			Mechanism of Action	Ref.
	J-Lat 9.2	J-Lat 8.4	J-Lat 10.6		
Panobinostat	0.10 ± 0.02	0.073 ± 0.010	0.041 ± 0.003	HDACi	[5,6]
Prostratin	7.1 ± 2.8	10 ± 1	1.8 ± 0.4	PKC activator	[5,6]
Psammaplin A	1.9 ± 0.3	1.5 ± 0.1	1.5 ± 0.1	HDACi	[29,30,31]
Aplysiatoxin	0.045 ± 0.021	0.011 ± 0.003	0.0033 ± 0.0012	PKC activator	[32,33,34]
Debromoaplysiatoxin	0.92 ± 0.14	0.52 ± 0.02	0.081 ± 0.029	PKC activator	[32,34]
Alotaketal C	1.3 ± 0.2	n.d.	n.d.	PKC activator	[14]
